# TNFSF10, an autophagy related gene, was a prognostic and immune infiltration marker in skin cutaneous melanoma

**DOI:** 10.7150/jca.86735

**Published:** 2023-07-31

**Authors:** Lei Xue, Wancong Zhang, Yikun Ju, Xuezheng Xu, Hao Bo, Xiaoping Zhong, Zhexiao Hu, Congyuan Zheng, Bairong Fang, Shijie Tang

**Affiliations:** 1 Department of Pathology, Hunan Cancer Hospital, The Affiliated Cancer Hospital of Xiangya School of Medicine, Central South University, Changsha, Hunan, China.; 2 Department of Plastic Surgery and Burn Center, Second Affiliated Hospital, Shantou University Medical College, Shantou, Guangdong, China.; 3 Plastic Surgery Institute of Shantou University Medical College, Shantou, Guangdong, China.; 4 Department of Plastic and Aesthetic (Burn) Surgery, The Second Xiangya Hospital, Central South University, Changsha, Hunan, China.; 5 Department of Orthopaedics, Hunan Cancer Hospital, The Affiliated Cancer Hospital of Xiangya School of Medicine, Central South University, Changsha, Hunan, China.; 6 NHC Key Laboratory of Human Stem Cell and Reproductive Engineering, Institute of Reproductive and Stem Cell Engineering, School of Basic Medical Science, Central South University, Changsha, Hunan, China.

**Keywords:** SKCM, autophagy, prognosis, immune infiltration, DNA methylation

## Abstract

Autophagy exerts a pivotal effect on skin cutaneous melanoma (SKCM). This study was aimed to investigate the expression of autophagy related genes (ARGs) in SKCM as well as its clinical value. Differentially expressed (DE) ARGs were downloaded from the intersection of SKCM data in GEPIA2 database and ARGs in Human Autophagy Database (HADB) database, and were verified in SKCM datasets GSE46517 and GSE15605. DE ARGs were enriched by Metascape online tools. According to GEPIA2 database, tumor necrosis factor-related apoptosis-inducing ligand (TNFSF10) was identified as a closely related factor and prognostic marker of SKCM. Then the correlation analysis of clinicopathological characteristics between TNFSF10 and SKCM was completed by several online tools such as TISCH, HPA, BEST and qRT-PCR. Subsequently, we investigated TNFSF10 related functions and signal pathways with LinkedOmics online tool, and immune infiltration using Assistant for Clinical Bioinformatics online tool. Furthermore, correlation analysis between TNFSF10 expression and immunotherapy response was performed by TIDE algorithm and BEST online tool. And Kaplan-Meier Plotter was used to assessing the prognosis of SKCM patients receiving immunotherapy. Finally, the correlation analysis among TNFSF10 methylation, TNFSF10 expression and patient prognosis was completed by the DiseaseMeth version 2.0, UCSC XENA and qRT-PCR. ARGs are DE in SKCM and participate in the ERBB signaling pathway, as well as the processing and presentation of antigens. Moreover, TNFSF10's expression along with methylation expression were significantly associated with the prognosis. Low expression of TNFSF10 was associated with malignant clinicopathological features, lower immune signal activity and lower immunocytes abundance in patients with SKCM. As an ARG, TNFSF10 has a potential capacity in predicting the prognosis of SKCM patients, meanwhile, may be a novel immunotherapy marker for SKCM.

## Introduction

As one of the most fatal skin malignancies, skin cutaneous melanoma (SKCM) originates from melanocytes of neural crest^1^,^2^. It is characterized by the high rates of recurrence, metastasis, mortality, and strong drug resistance^3^. Recently, the incidence of SKCM has been increasing^4^. In the US, the incidence of SKCM ranked fifth and sixth in male and female tumors, respectively^5^. It was estimated that there were 287,700 new cases of melanomas, resulting in 60,700 deaths worldwide in 2018^6^. Moreover, survival outcomes of patients within the same stage even can be greatly different from each other because of roles of effector T cells specific for the melanoma antigens varies in mediating antitumor immunity^2^,^7^. To date, prognostic prediction still mainly relies on histopathological diagnosis and tumor staging system. However, such traditional approaches might not be sufficient for evaluating precisely the outcomes of SKCM patients. Thus, it is indispensable to identify as well as develop the biomarkers of prognosis for risk stratification along with treatment optimization innovatively among patients with melanomas^4^,^8^. Exploring biomarkers associated with tumor progression and prognosis has important clinical significance and research value^9^.

Autophagy, as a survival-promoting pathway, captures and degrades intracellular components such as proteins and organelles in lysosomes^10^,^11^. In normal cells & tissues, autophagy is basally active to keep cellular homeostasis along with the quality of protein and organelle through the elimination of impaired organelles as well as protein aggregates^11^. Autophagy is closely related to tumor. However, it is so complex that it will even show the opposite biological and clinical effects, depending on different cancer types and genetic background^12^,^13^. Wang et al.^14^ revealed the important role of autophagy-related gene (circROBO1) in promoting tumorigenesis, progression and metastasis. Although autophagy inhibits tumorigenesis sometimes, it promotes tumorigenesis in the majority of the cases. In response to elevated energetic demands that arises from rapid proliferation and inadequate blood supply, a high basal autophagy gives a metabolic advantage to tumours^15^, which will not only help tumors to survive microenvironmental pressure, but also increase their growth and invasiveness. Therefore, to improve cancer treatment, inhibiting autophagy has aroused great interest^10^. Autophagosome's formation requires sequential actions of autophagy-related genes (Atg) proteins, which was a unique set of autophagy mediator proteins, producing two ubiquitin-like conjugation systems of the Atg5-Atg12-Atg16 conjugation system as well as the LC3 conjugation system to convert its cytosolic form (LC3-I) into its lapidated form (LC3-II), or to incorporate LC3-II into the autophagosome membranes^16^,^17^.

Increasing studies have demonstrated the implication of autophagy in melanoma. Acquired resistance to BRAF inhibitors for melanoma has been found to be linked to endoplasmic reticulum (ER) stress-associated autophagy induction, at the same time, it has been put forward that BRAF inhibitor-induced autophagy might be a therapeutic target for the melanomas 18. The inhibition of autophagy by targeting mTOR and AMPK could potentiate the cytotoxicity effects of PA on melanoma cells^19^. Furthermore, some researchers suggested that the status of ATG5 and autophagy could serve as diagnostic markers for distinguishing the malignant of melanocytes from benign tumors^20^. Additionally, some researchers have also suggested that targeting RIPK1's autophagy-activating mechanism is possibly to be helpful to upregulate the melanoma cell's sensitivity to therapeutic agents inducing ER stress^21^. Previously, autophagy's effect on the progression as well as treatment of melanomas have attracted a lot of attention, however, its prognostic role in melanomas was rarely studied. Moreover, the genome landscape of ARGs is not clear.

In this article, we used data mining to probe the role of ARGs in SKCM. We found Tumor necrosis factor-related apoptosis-inducing ligand (TNFSF10), an ARG, was linked with SKCM patients' prognosis of, furthermore, significantly related to the immune infiltration in SKCM. Previous studies have confirmed that TNFSF10 is a p53-transcriptional target gene^22^. Silencing of TNFSF10 gene can inhibit the activity of caspases 3 and caspases 7^22^. This broadens the relationship between autophagy and immunity, and enriches our understanding about the pathogenesis of SKCM.

## Materials and Methods

### Differential ARGs screening, validation, and functional enrichment analysis

The differential expression analysis of the TCGA SKCM data set is performed by the GEPIA2 (http://gepia2.cancer-pku.cn/#index) online tool^23^, and the screening threshold is |log2FC|>1 & Q-value < 0.01. A total of 231 ARGs were downloaded from Human Autophagy Database (HADb) (http://www.autophagy.lu/index.html). The HADb has been developped in the Laboratory of Experimental Cancer Research headed by Dr Guy Berchem. Intersection analysis as well as heat map clustering analysis were all carried out by ImageGP online tool^24^. SKCM microarray data (GSE46517 and GSE15605) were obtained from Gene Expression Omnibus (GEO) database (https://www.ncbi.nlm.nih.gov/geo/)^25^. The functional enrichment analysis of differentially expressed (DE) ARGs was conducted by Metascape online tools (http://metascape.org/gp/index.html#/ main/step1)^26^.

### Correlation analysis between ARGs and patient prognosis

The correlation analysis between DE ARGs and patient prognosis is carried out by GEPIA2 online tool based. TNFSF10 expression data, methylation data, prognostic data, etc. in the TCGA SKCM cohort were all downloaded from the UCSC XENA database (https://xena.ucsc.edu/)^27^ and microarray data sets of SKCM were downloaded from the GEO database. Log-rank p test was performed to analyze the relationship of TNFSF10's expression or methylation level with the prognosis of SKCM patients. The relevant graphics were analyzed and drawn by the online tools Best (https://rookieutopia.com/app_direct/BEST/), Kaplan Meier plotter (http://kmplot.com/analysis/)^28^ and UCSC XENA based on GSE133713, GSE46517, GSE98394, GSE65904 and TCGA SKCM cohort data.

### The expression of TNFSF10 in SKCM as well as its association with clinicopathological features of patients

The expression of TNFSF10 in SKCM patients with different clinicopathological features in the data sets GSE46517, GSE98394, GSE190113 and TCGA_SKCM has been analyzed by the BEST online tool. Based on clinicopathological stages (Tumor-node-metastasis stage, TNM stages), ages and genders. The expression of TNFSF10 in different cells in the SKCM single-cell RNA sequencing data sets GSE115978 and GSE72056 has been analyzed by the TISCH (http://tisch.comp-genomics.org/home/) online tool^29^. The expression data of TNFSF10 protein in normal skin and SKCM were got from the Human Protein Atlas database (https://www.proteinatlas.org/)^30^.

### Cell culture, clinical sample collection and qRT-PCR

The A375 cell line was obtained from the Department of Dermatology, Xiangya Hospital, Central South University, and cultured as previously described^31^. Clinical samples were collected from The Affiliated Cancer Hospital of Xiangya School of Medicine, with a total of 24 cases of melanoma and 8 cases of normal tissues. The fresh tissues were stored at liquid nitrogen. This study was conducted in accordance with the Declaration of Helsinki. All tissues were ground and extracted the RNA, then qRT-PCR detected. The following primers were used for qRT-PCR: TNFSF10, 5′-AGTCAAGTGGCAACTCCGTC-3′ and 5′-AGTCAAGTGGCAACTCCGTC -3′; ACTB, 5′-TCACCAACTGGGACGACATG-3′ and 5′-GTCACCGGAGTCCATCACGAT-3′.

### TNFSF10 related functions and signal pathway enrichment analysis

TNFSF10 related genes analysis and signal pathway enrichment analyses were completed by LinkOmics online tool (http://www.linkedomics.org/login.php) based on the TCGA SKCM cohort data according to the default parameter^32^. Spearman correlation analysis was adopted, and the screening threshold is FDR<0.01.

### Correlation analysis of the level of TNFSF10 expression with immune infiltration among SKCM patients

We analyzed the correlation of the level of TNFSF10 expression with immunocytes abundance and immune related genes expression in TCGA SKCM cohort data using Assistant for Clinical Bioinformatics (https://www.aclbi.com/static/index.html#/) based on TIMER algorithm 33. We collected tumor samples from eight melanoma patients and evaluated the correlation between TNFSF10 (1:100) and PDCD1 (1:100) by immunohistochemical staining. Immunohistochemical staining was performed according to the kit. TNFSF10 Antibody was purchased from the ABclonal company. PDCD1 Antibody was purchased from the MXB Biotechnologies company. Ethical approval was obtained for the parts of this experiment involving human samples, and informed consent was obtained from the patients.

### Statistical analysis and drawing

The difference between the two groups was analyzed by Student's t test, and the difference between multiple groups was analyzed by analysis of variance (ANOVA). All graphs in this study were drawn by online tools or GraphPad software. P or FDR value less than 0.05 was statistically significant.

## Results

### ARGs were DE and functional in SKCM

**Figure 1:**
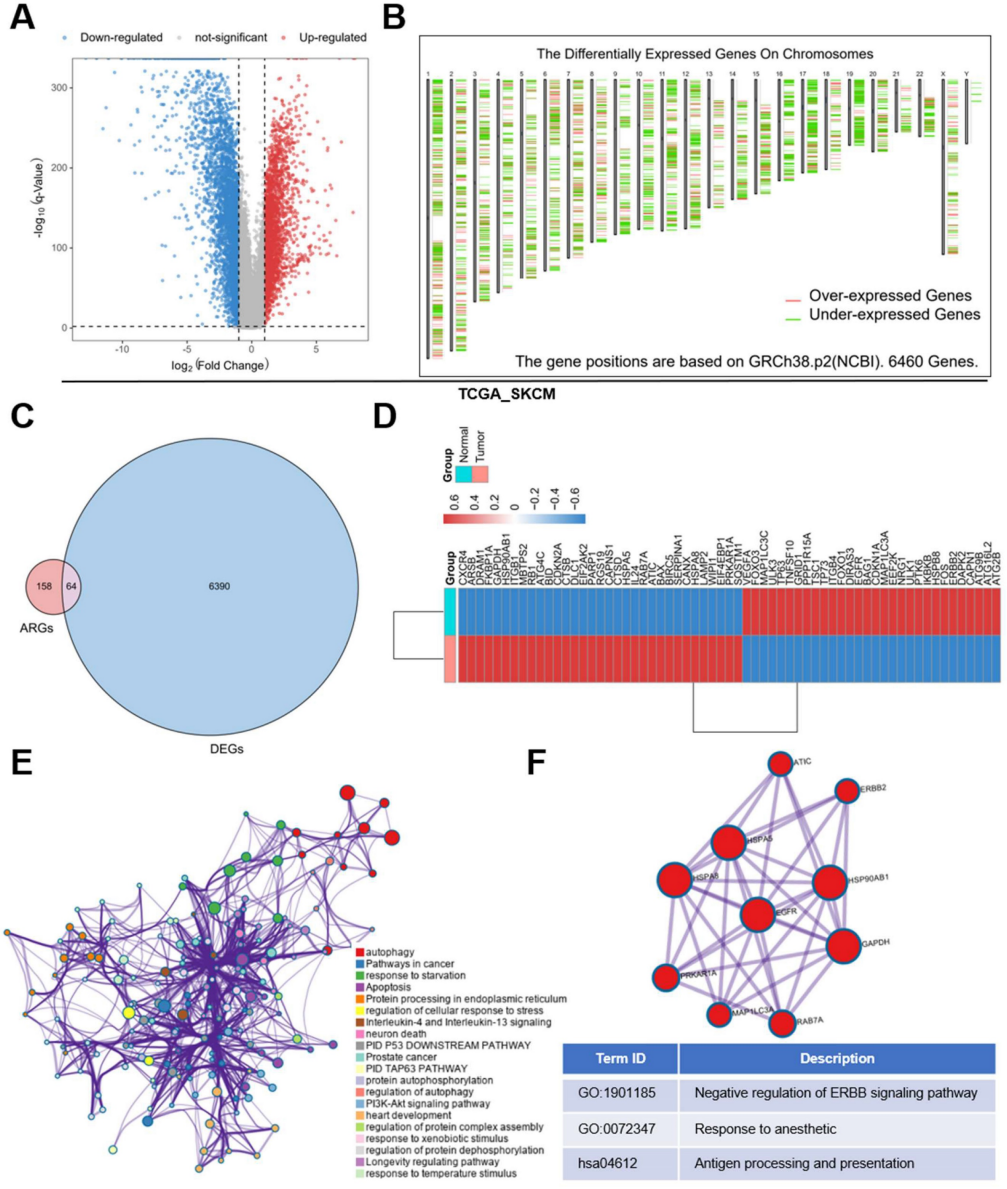
**DE ARGs screening and functional enrichment analyses.** (A) Volcano plot of 6460 significant DE genes. (B) Distribution of DE genes on chromosomes. (C) 232 genes from HADB were crossed with 6460 significant genes, and 64 DE ARGs were obtained. (D) 34 up-regulated ARGs and 30 down-regulated ARGs were shown by heatmap. (E) Network of enriched items with p-value < 0.05. (F) The core module of the network.

First, we obtained 6460 DE genes from TCGA SKCM dataset in using GEPIA2 database **(Figure 1A, 1B)**. Meanwhile, 232 ARGs were obtained from HADB, after which intersection was taken between the two gene sets **(Figure 1C)** and 64 DE ARGs were identified (34 up-regulated; 30 down-regulated) **(Figure 1D)**. To verify these DE ARGs, we downloaded two datasets of SKCM microarray data (GSE46517 and GSE15605), and selected randomly three up-regulated and three down-regulated ARGs samples for verification, from which we found that results were basically consistent with those of TCGA **(Supplementary Figure 1A, 1B, 1C, 1D)**.

To explore the function of these genes, these DE molecules were submitted to Metascape online tools for Gene ontology (GO) analysis as well as Kyoto Encyclopedia of Genes and Genomes (KEGG) analysis. The analysis revealed that besides the regulation of autophagy, the DE ARGs were mostly enriched when responding to starvation, temperature stimulus and so on in GO. And the metabolism-related pathways like PI3K-Akt signaling pathway, apoptosis and immune related pathways like Neutrophil degranulation and Neurotrophin signaling pathway were enriched in KEGG** (Figure 1E)**. In addition, we found a core module in the network, which revealed that these DE ARGs were mostly involved in erythroblastic leukemia viral oncogene homolog (ERRB) signaling pathway, response to anesthetic, antigen processing and presentation **(Figure 1 F)**.

### TNFSF10 might be the best prognostic markers of SKCM

**Figure 2:**
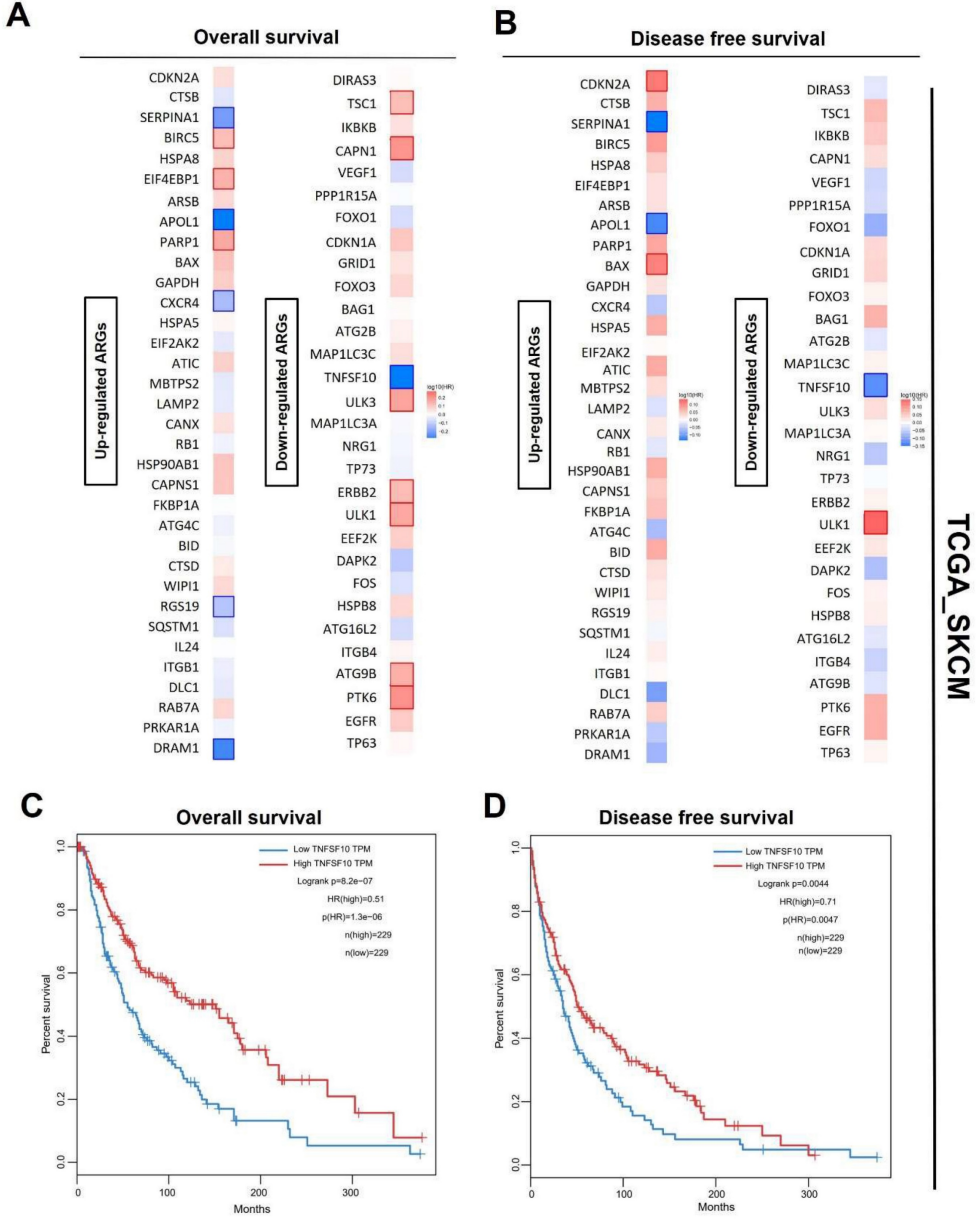
** Prognosis analysis of DE ARGs in SKCM**. (A) The OS hazard ratio heatmap of 64 DE ARGs. (B) The disease-free survival hazard ratio heatmap of 64 DE ARGs. (C) The relationship of TNFSF10's expression level with SKCM patients' Overall survival. (D) The relationship of TNFSF10's expression level with SKCM patients' Disease-free survival.

Also, we analyzed the prognosis (overall survival [OS]/ recurrence-free survival [RFS]) of SKCM patients of these differentially-expressed ARGs with a median cutoff using GEPIA2. The heatmaps showed the risk ratio (HR of OS/RFS) of each ARG. Interestingly, the result revealed that only TNFSF10 was significantly associated with both OS and RFS among all the ARGs** (Figure 2A, 2B)**. OS and disease-free survival analysis in SKCM also confirmed this finding, suggesting TNFSF10 as a vital prognostic marker in SKCM patients **(Figure 2C, 2D)**. Prognostic analysis based on the data from the other independent SKCM cohorts based on the GEO data sets (GSE133713, GSE46517, GSE98394, GSE65904) also showed that TNFSF10 might be a valuable marker** (Supplementary Figure 2A-F)**.

### The down-regulation of TNFSF10 expression in SKCM was associated with the clinicopathological characteristics of patients

**Figure 3:**
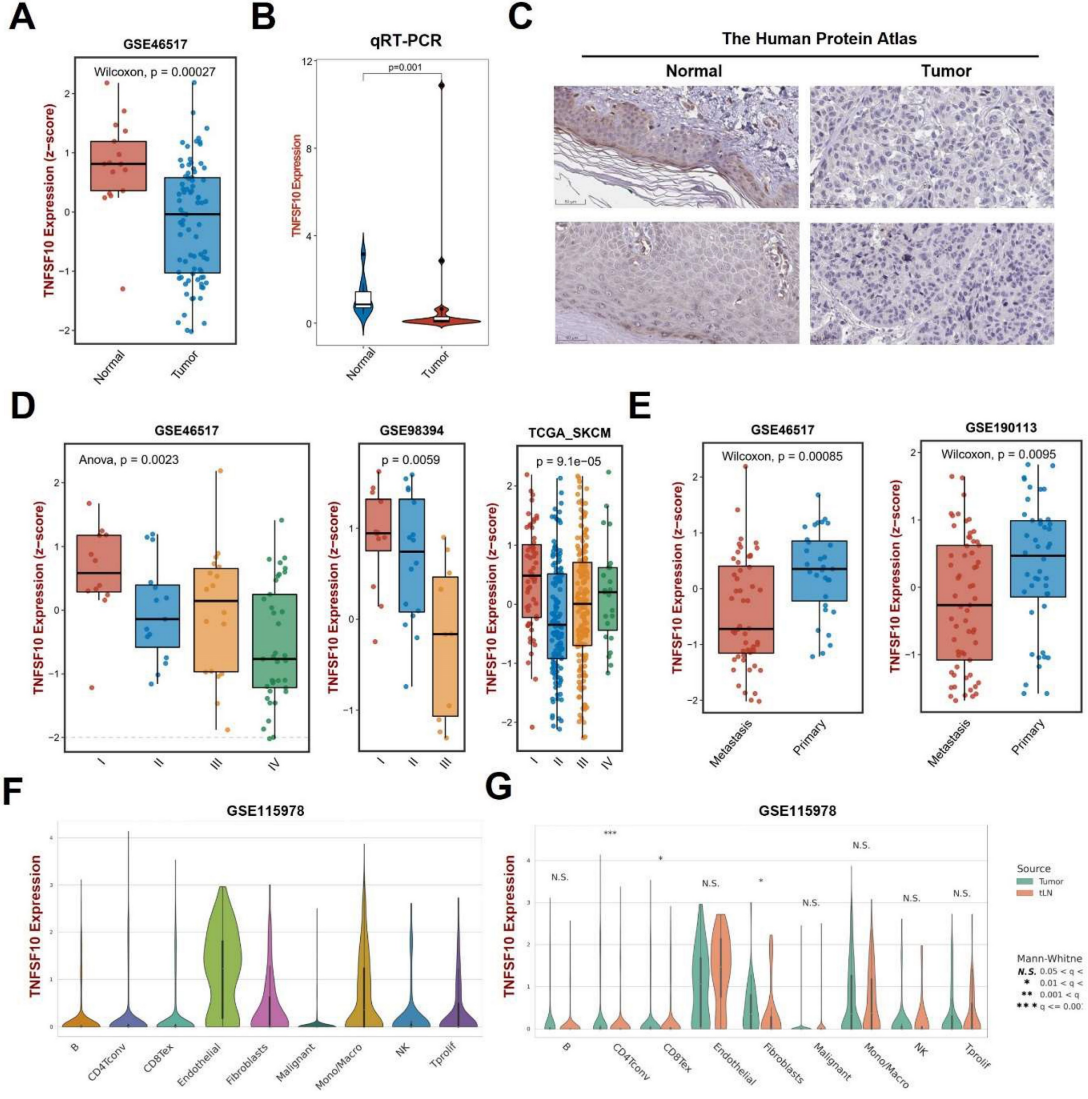
** The expression of TNFSF10 in SKCM as well as its correlation with the clinicopathological characteristics of patients.** (A) TNFSF10 expression in normal skin and SKCM tissues base on GSE46517. (B) qRT-PCR verified the expression of TNFSF10 in normal skin and SKCM tissues in clinical samples. (C) TNFSF10 protein expression in normal skin and SKCM tissues from the Human Protein Atlas database. Scale is 50μm. (D) Differences in the expression of TNFSF10 in different SKCM stages based on multiple data sets. (E) Differences in the expression of TNFSF10 in primary and metastasis tumor based on multiple data sets. (F & G) TNFSF10 expression in different cell types from SKCM single-cell RNA sequencing data.

We analyzed the expression of TNFSF10 and found that its expression was also lower in SKCM tumors than normal tissues on GSE46517** (Figure 3A)**. Then, we verified it by clinical samples using qRT-PCR and found consistency with previous analysis results **(Figure 3B)**. We also analyzed the expression of TNFSF10 protein and found that its expression was also lower in SKCM **(Figure 3C).** Our analysis based on multiple datasets showed that TNFSF10 expression levels were lower in patients with advanced SKCM stages** (Figure 3D)**. TNFSF10 expression levels were also lower in older patients older than 65 years of age, those patients tend to have a worse prognosis **(Supplementary Figure 3A)**. Also, we compared the different expression of TNFSF10 between metastasis tumor and primary tumor. The results showed that TNFSF10 expression levels were lower in metastasis tumor than primary **(Figure 3E)**. Consistent with expression analysis of TNFSF10 in TNM stages, TNFSF10 expression levels was lower in M1 stages **(Supplementary Figure 3B)**. Lymph node metastases are also associated with TNFSF10 expression, TNFSF10 expression was higher in N0 stages (without lymph node metastases) **(Supplementary Figure 3C)**. Furthermore, we analyzed single-cell RNA sequencing data of SKCM, and found that TNFSF10 was expressed in almost all cell types of SKCM **(Figure 3F, 3G and Supplementary Figure 3D)**. Additionally, TNFSF10's expression was lower in CD4+ T cells, CD8+ T cells and fibroblasts of SKCM with lymph node metastasis. However, the difference among these three types of cells in patients of different genders was not significant** (Supplementary Figure 3E)**.

### TNFSF10 might participate in multiple immune signal pathways in SKCM

**Figure 4:**
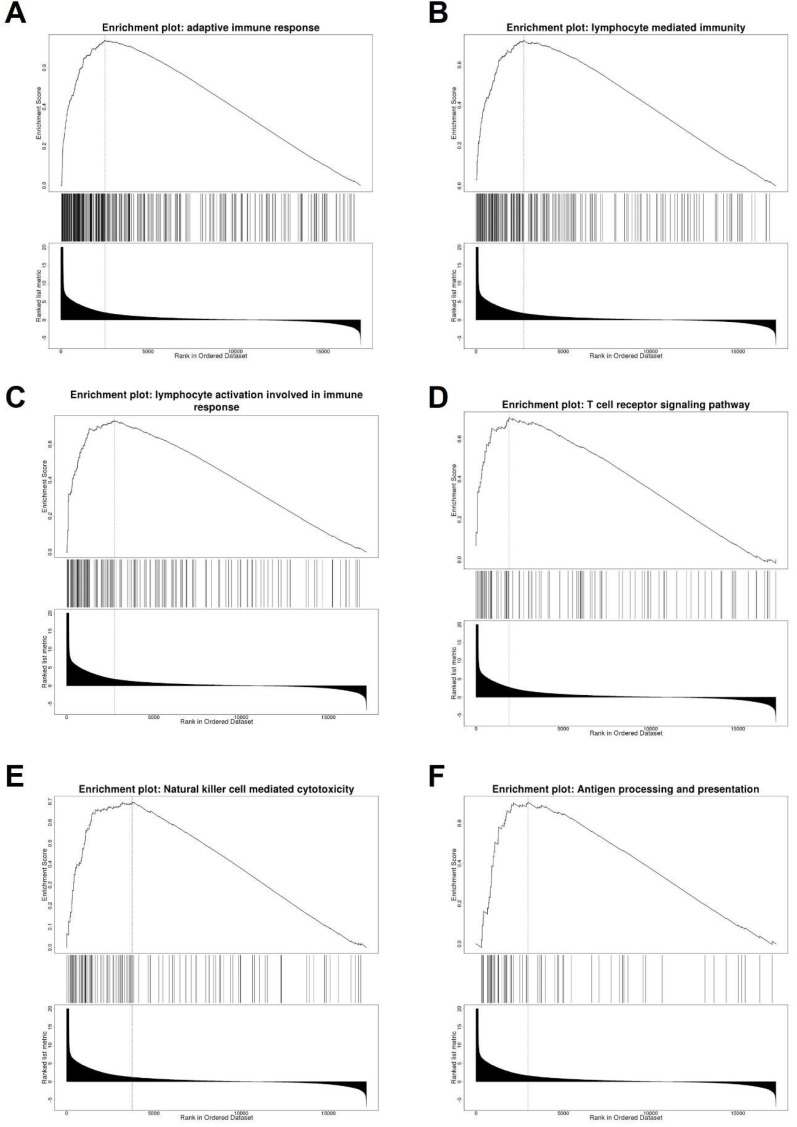
** Analysis of TNFSF10 related genes and related signal pathways.** (A-F) GSEA enrichment analysis of TNFSF10 related signal pathway.

To explore the possible function of TNFSF10 in SKCM, we searched for genes related to TNFSF10 expression** (Supplementary Figure 4)**, and displayed the top 50 correlated genes with heatmaps either positively or negatively, found that there are several immune-related genes, such as CXCL1, CXCL9 **(Supplementary Figure 5)**. Furthermore, we carried out a GSEA enrichment analysis, suggesting that the adaptive immune response, lymphocyte-mediated immunity, T cell receptor signaling and other signaling pathways were significantly enriched **(Figure 4A-F)**. It suggested that TNFSF10 may exert a vital effect on the anti-tumor immune process of SKCM.

### TNFSF10 expression was closely related to immune infiltration in SKCM

**Figure 5:**
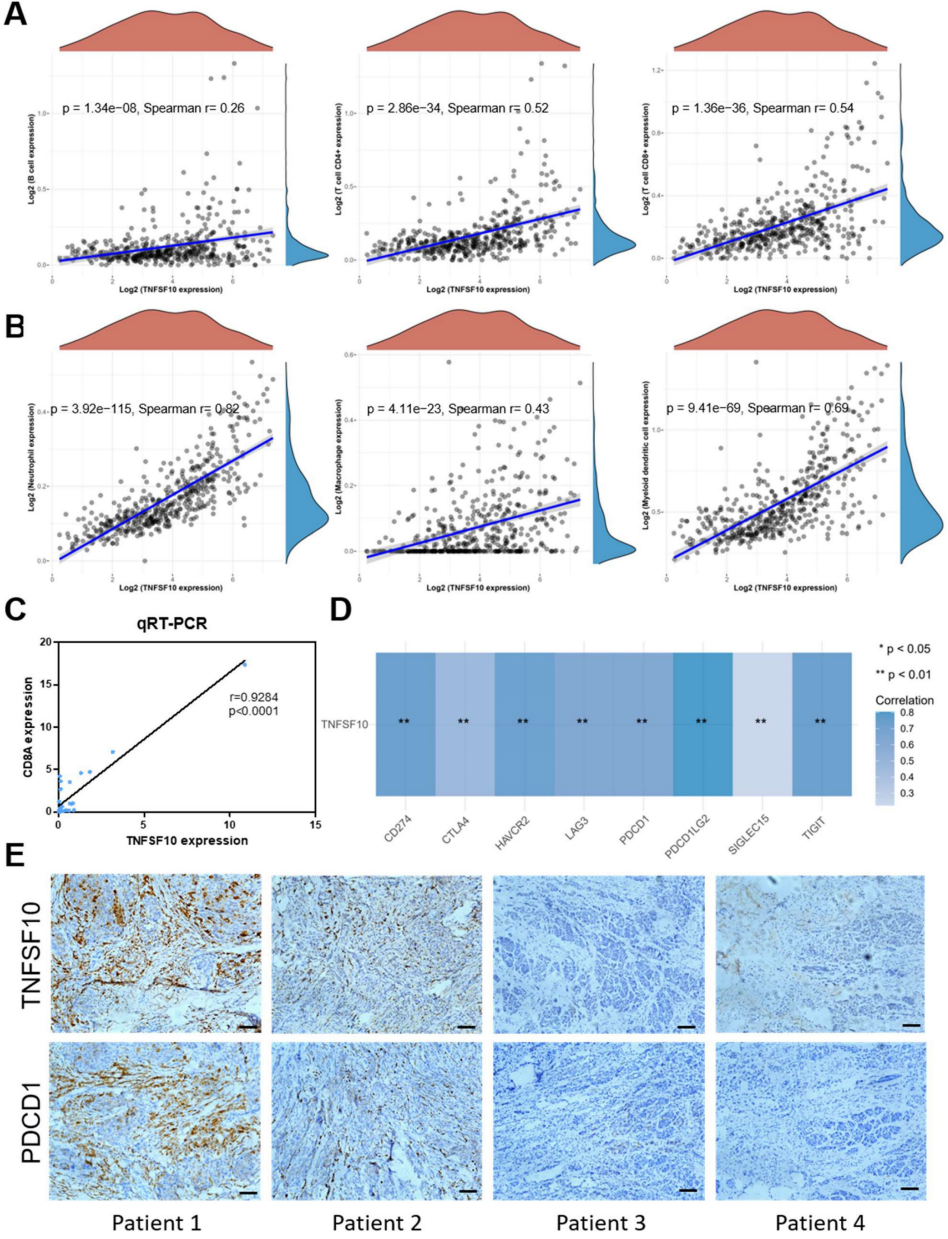
** TNFSF10 expression was closely related to immune infiltration in SKCM.** (A) The correlation between TNFSF10 expression and the abundance of B cells, CD4+ T cells, CD8+ T cells. (B) The correlation among TNFSF10 expression and the abundance of Neutrophil, Macrophage, Myeloid dendritic cell. (C)The correlation between TNFSF10 expression and CD8A expression in SKCM verified by qRT-PCR. (D) The correlation among TNFSF10 expression and the expression of immune checkpoint molecules in SKCM. (E) Representative immunohistochemical staining images of TNFSF10 and PDCD1. Scale is 100μm.

We analyzed the association of TNFSF10's expression with immunocytes abundance among TCGA SKCM cohort data. As a result, it was found that TNFSF10 was positively related to immunocytes abundance of B cells, CD4 + T cells, CD8 + T cells with statistical significance **(Figure 5A)**, Neutrophil, Macrophage, Myeloid dendritic cells** (Figure 5B)**, most of which were with r>0.6. The results of qRT-PCR based on clinical samples showed that CD8A expression was positively correlated with TNFSF10 expression **(Figure 5C)**. CD8A is an important leukocyte differentiation antigen that is expressed on T cells. Moreover, TNFSF10 was positively correlated with most of the members of immune checkpoint molecules in SKCM such as PDCD1LG2, CD274, HAVCR2, TIGIT, etc.** (Figure 5D)**. Consistent with these findings, we also found that patients with high TNFSF10 expression had higher abundance of immunocytes such as CD8+ T cells, CD4+ T cells, B cells, etc. **(Supplementary Figure 6A)**, and higher expression of immune checkpoint molecules** (Supplementary Figure 6B)**. We collected samples from 8 melanoma patients for immunohistochemical staining. The correlation of TNFSF10 with immune checkpoint-related genes was assessed by staining for TNSF10 and PDCD1. The results showed a strong correlation between TNFSF10 and PDCD1, and we show typical immunohistochemical staining images of 4 cases **(Figure 5E)**.

### TNFSF10 might be a potential biomarker for SKCM's immunotherapy

**Figure 6:**
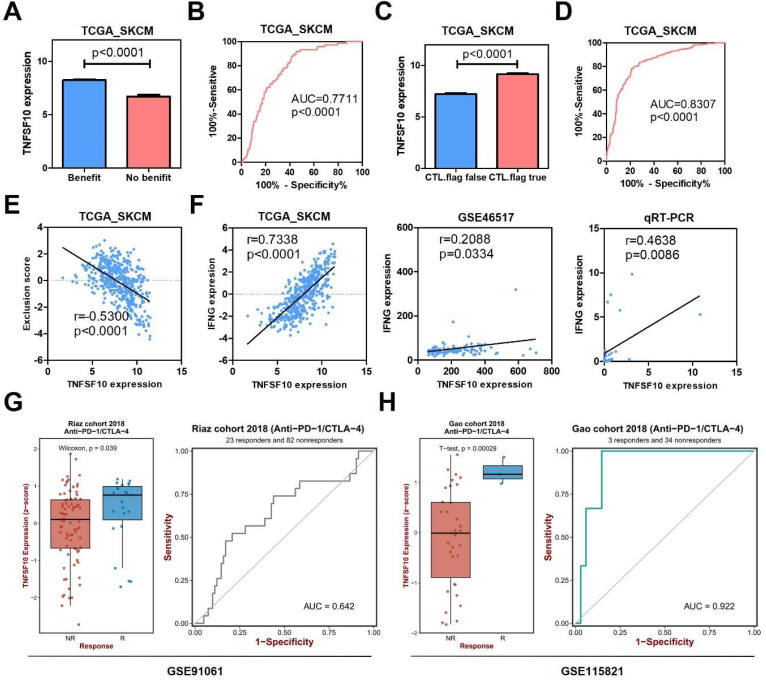
** The value of TNFSF10 as a marker of immunotherapy response in SKCM.** (A) The expression difference of TNFSF10 in SKCM patients who can/cannot benefit from immunotherapy. (B) The diagnostic efficacy of TNFSF10 expression in judging whether SKCM immunotherapy is beneficial. (C) The expression difference of TNFSF10 in the SKCM tissues of cytotoxic lymphocyte flag positive and negative. (D) The expression of TNFSF10 was effective in diagnosing whether cytotoxic lymphocyte flag would appear after immunotherapy in patients with SKCM. (E) Correlation between the expression of TNFSF10 and the Exclusion score. (F) Correlation between the expression of TNFSF10 and the expression of cytotoxic lymphocyte effector molecule IFN. (G & H) The expression difference of TNFSF10 in Anti-PD-1/CTLA-4 therapy responders and nonresponders based on different data sets.

**Figure 7:**
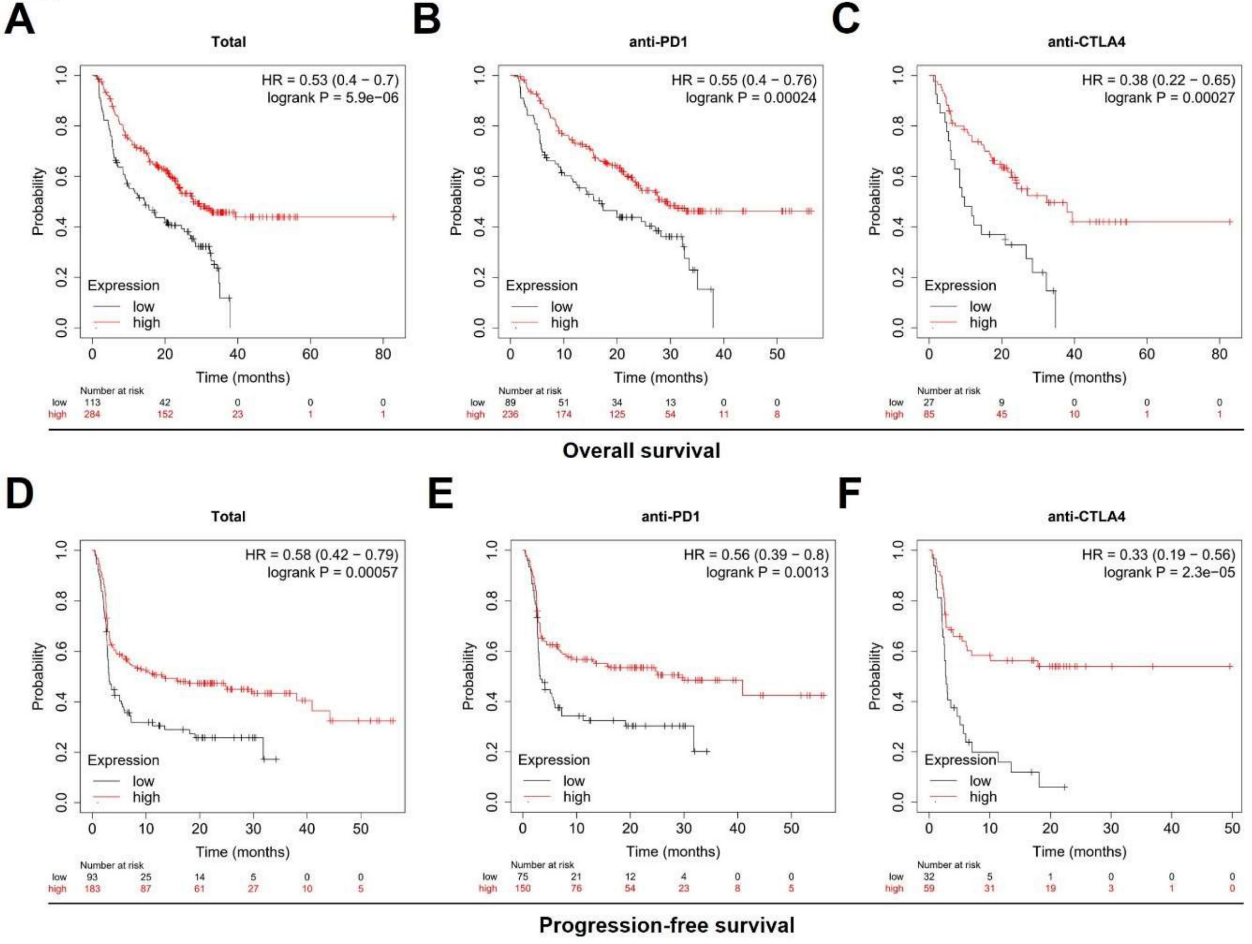
** High TNFSF10 expression predicts a better prognosis for SKCM patients receiving immunotherapy.** (A, B, C) The relationship of TNFSF10's expression level with SKCM patients' Overall survival after immunotherapy (Total, anti-PD1, anti-CTLA4). (D, E, F) The relationship of TNFSF10's expression level with SKCM patients' Progression-free survival after immunotherapy (Total, anti-PD1, anti-CTLA4).

In order to further explore the clinical value of TNFSF10 in anti-tumor immunity, we used the TIDE algorithm for predictive analysis, and we found that the expression of TNFSF10 was higher in patients who could benefit from SKCM immunotherapy. Thus, based on the expression of TNFSF10** (Figure 6A)**, it was possible to distinguish whether patients with SKCM could benefit from immunotherapy** (Figure 6B)**. In addition, we also found that patients with CTL flag true in SKCM tissue had higher expression of TNFSF10 **(Figure 6C)**. Thus, based on the expression of TNFSF10, it was also possible to distinguish whether the CTL flag true or not** (Figure 6D)**. The expression of TNFSF10 was negatively correlated with the Exclusion score **(Figure 6E)**. Analyze based on two data sets showed that the expression of TNFSF10 was also positively linked to the effector molecule IFN's expression of cytotoxic lymphocytes, and verified it by qRT-PCR with clinical samples **(Figure 6F)**. Consistent with this, the expression of TNFSF10 in Anti-PD-1/CTLA-4 therapy responders was higher than nonresponders **(Figure 6G, 6H)**. Importantly, high TNFSF10 expression indicates that SKCM patients can benefit more from immunotherapy either anti-PD1 or anti-CTLA4 immunotherapy **(Figure 7A-F)**. Those SKCM patients with high TNFSF10 expression had higher overall survival and progression-free survival after immunotherapy. These data showed that TNFSF10 might be an important marker of the response to immunotherapy in SKCM patients.

### Negative correlation between TNFSF10 methylation in SKCM

**Figure 8:**
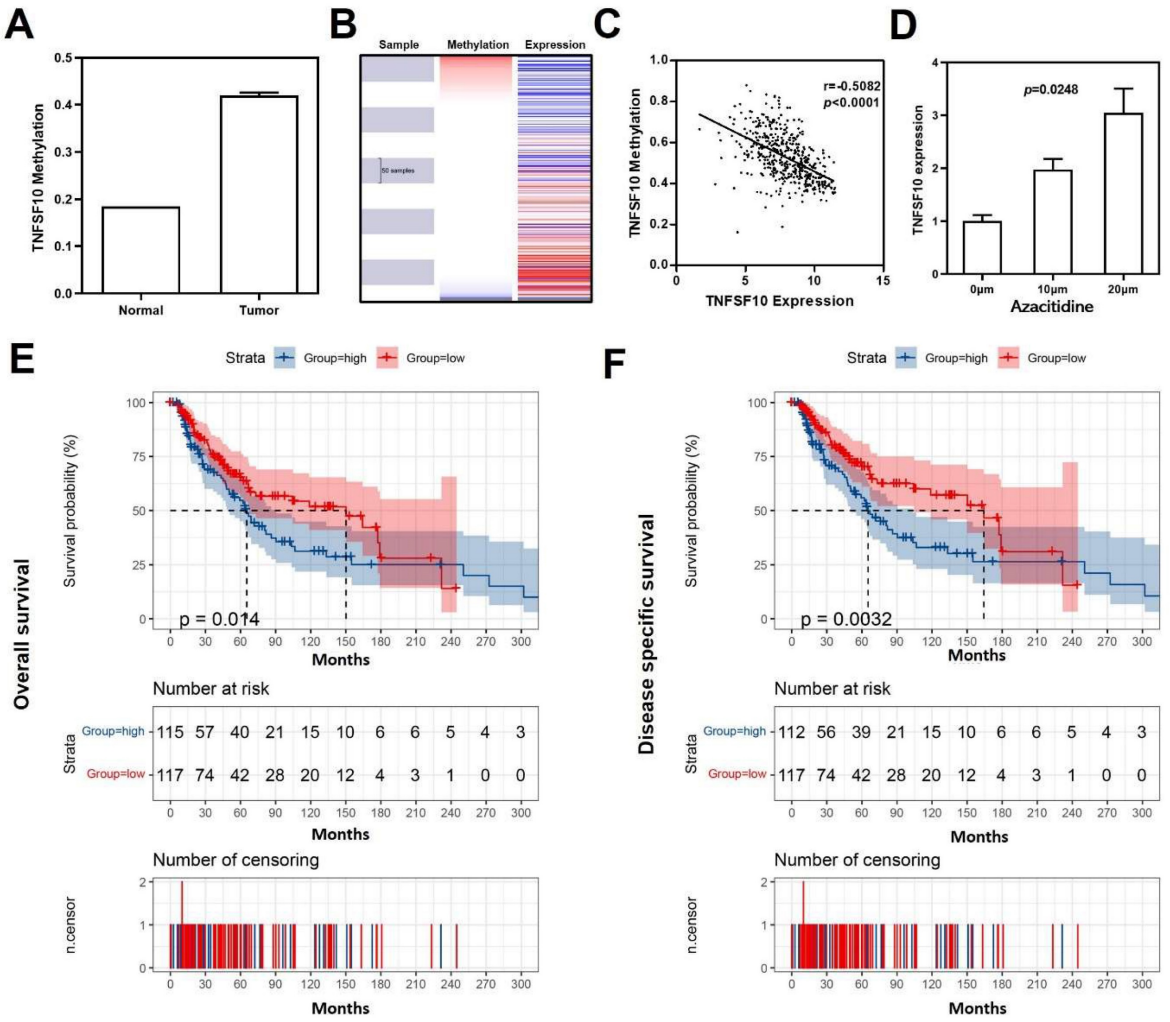
** Methylation analysis of TNFSF10 in SKCM.** (A) The methylation level of TNFSF10 in SKCM as well as normal tissues. (B-C) The correlation of TNFSF10's methylation level with the prognosis of SKCM. (D) Changes of TNFSF10 expression in A375 cell line after azacytidine treatment. (E, F) The prognosis analysis of TNFSF10 methylation in SKCM.

As reported, DNA methylation acts as one of the most critical factors when regulating gene expression^34^. Previous result has demonstrated significant down-regulation of TNFSF10 in SKCM, so we further analyzed the average methylation level of TNFSF10 promoter region in SKCM using DiseaseMeth version 2.0 online database and found that TNFSF10 was significantly hypermethylated in SKCM **(Figure 8A)**. Analogously, a negative correlation with statistical significance was discovered between TNFSF10's methylation and expression levels based on UCSC XENA online tool **(Figure 8B, 8C)**. We treated the A375 cell line with azacytidine (10μm and 20μm) and determined the difference in TNFSF10 expression between the administration and the blank control group using qRT-PCR** (Figure 8D)**. The results showed the expression of TNFSF10 was higher in the administration group than the blank control group. The prognostic significance of TNFSF10 methylation level was also analyzed using the tool, from which we found TNFSF10 methylation level was negatively associated with the prognosis (OS/DSS) of SKCM** (Figure 8E, 8F)**.

## Discussion

Over the past decade, our understanding of melanoma has been improved tremendously. In the molecular pathogenesis of diseases, the mutations allowing for tumorigenesis, and the interactions of the autophagy with immune system allowing for tumor survival or distant metastasis has become more and more explicit 18,35,36. As reported recently, the prognosis of SKCM has been predicted by the construction of a prognostic signature based on exosomes along with genes^37^,^38^, however, the autophagy-related prognostic signature to predict the SKCM's prognosis was few constructed, and the possible role of ARGs in SKCM was rarely explored bioinformatically. In addition, we also performed enrichment analysis and PPI analysis on the DE ARGs, and found that the immunity correlating pathways were enriched, such as autophagy, apoptosis, PI3K, interleukin and antigen presentation. It is suggested that there exists a complex interaction between autophagy and immunity in SKCM.

The prognosis of patients with cancer is closely related to autophagy. Thus, to find a prognostic signature for SKCM patients is especially meaningful. Besides, we also utilized GEPIA2 to explore the correlation between 64 ARGs and the prognosis (OS/RFS) of SKCM. We found that only TNFSF10 could be associated with OS and RFS. TNFSF10, either acting as one member of TNF-α family or a death receptor ligand, was discovered to have the capacity of selectively killing tumor cells 39. TNFSF10 could trigger apoptosis in a cell-autonomous way among various cell lines of tumors 40. Experiments (in vivo and in vitro) revealed that TNFSF10 was a melanoma metastasis-related gene, which may lie the foundation of the development of novel, molecularly targeted drugs^41^,^42^. Our study first showed that TNFSF10 is a good prognostic marker for SKCM.

Immunotherapy has become a leading paradigm for the treatment of advanced melanoma. Current therapies include systemic cytokines, immune checkpoint inhibitors (ICIs), as well as localized intratumoral therapies. ICIs can block the natural pathways, including programmed cell death 1 receptor (PD-1)/programmed death-ligand 1 (PD-L1) as well as cytotoxic T lymphocyte antigen-4 (CTLA-4), to inhibit the immune response to stimuli. Not only in the monotherapy but also in combination therapy, the effectiveness of TNFSF10 has been proved in clinical systemic immunotherapies^43^. TNFSF10 is also intimately involved in anti-cancer surveillance through immune cells^44^,^45^, which is consistent with our results. Based on our data, we speculate that TNFSF10 may not only regulate the autophagy, but also activate the expression of immune-related genes and participate in anti-tumor immune regulation. Interestingly, we found for the first time that TNFSF10 had high sensitivity and specificity in predicting the benefit of immunotherapy among SKCM patients. Thus, TNFSF10 has the capacity as a molecular marker of the response to immunotherapy in SKCM patients. TNFSF10 is a death ligand cytokine that is used primarily by effector immune cells to kill malignant transformed cells^22^,^46^. It has been proposed that nanotechnology can be used to induce autophagy in tumor cells by targeting TNFSF10 for tumor treatment^46^. Meanwhile, autophagy therapy can enhance the sensitivity of tumors to immunotherapy and play a certain synergistic role^47^. Studies on inflammatory and autoimmune models have shown that TNFSF10 can affect immune cells in many different ways^48^. TNFSF10 is expected to be effective in controlling tumors with minimal side effects^48^,^49^.

DNA methylation, an epigenetic variation, which may mediate the pathogenic activity of tumor. As an epigenetic hallmark of melanoma, aberrant DNA methylation is known to be pivotal for the formation as well as progression of melanomas 50. Recently, advanced methods in genome-wide methylation have provided ways for the identification of differentially methylated genes, methylation signatures, along with potential biomarkers. Despite of massive effort as well as progresses achieved in cataloging the changes of methylation in melanomas, several issues have remained unresolved^51^. Our findings indicated that DNA methylation would lead to lower expression of TNFSF10 in SKCM. The studies showed that DNA methylation of TNFSF10 was an important factor in the pathogenesis of immune system^52^. This result may contribute to speeding up the utilization of the potential capacity of DNA methylation of TNFSF10 in the diagnosis, prognosis, as well as therapeutic effect, which will provide significant benefit for these patients.

In a word, through analysis of bioinformatics, we found that TNFSF10, an autophagy-related gene, was highly valuable as a marker of the prognosis of SKCM patients. At the same time, it may also be a potential biomarker of the response to immunotherapy in SKCM patients. Further studies on TNFSF10's role and mechanism in SKCM will bring new ideas to the diagnosis and treatment of SKCM.

## Supplementary Material

Supplementary figures.
