# Supplementary material for: TNFSF10, an autophagy related gene, was a prognostic and immune infiltration marker in skin cutaneous melanoma
Source: J Cancer. 2023 Jul 31;14(13):2417–30. doi: 10.7150/jca.86735 (PMC10475358; doi:10.7150/jca.86735)
Supplement: Supplementary file 1 — Supplementary figures. [file jcav14p2417s1.pdf]

Supplementary Material

Supplementary Figure 1

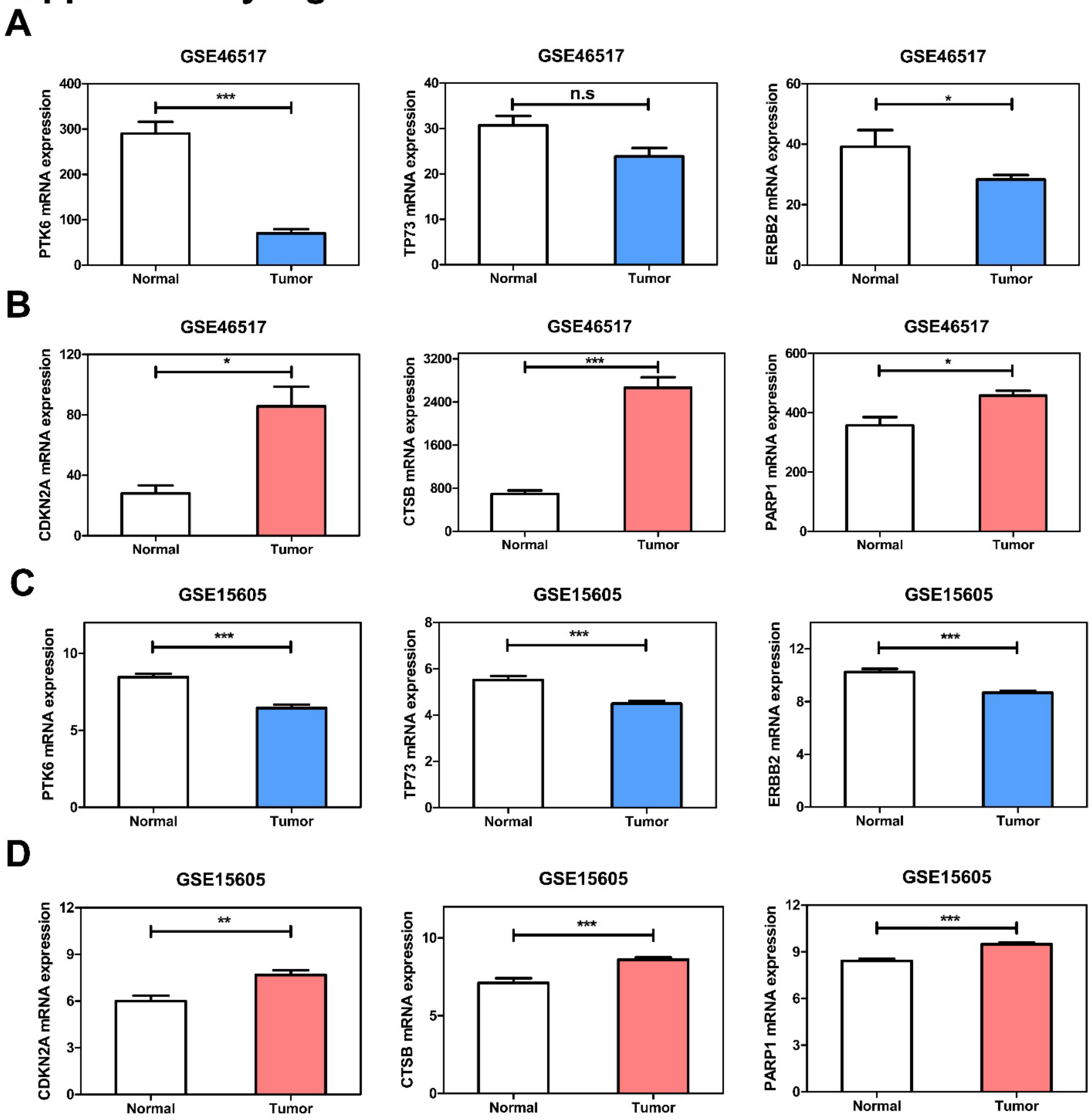

Supplementary Figure 1. Verification the DE ARGs. (A & B) Expression of randomly selected genes in GSE46517 dataset. (C & D) Expression of randomly selected genes in GSE15605 dataset.

Supplementary Figure 2

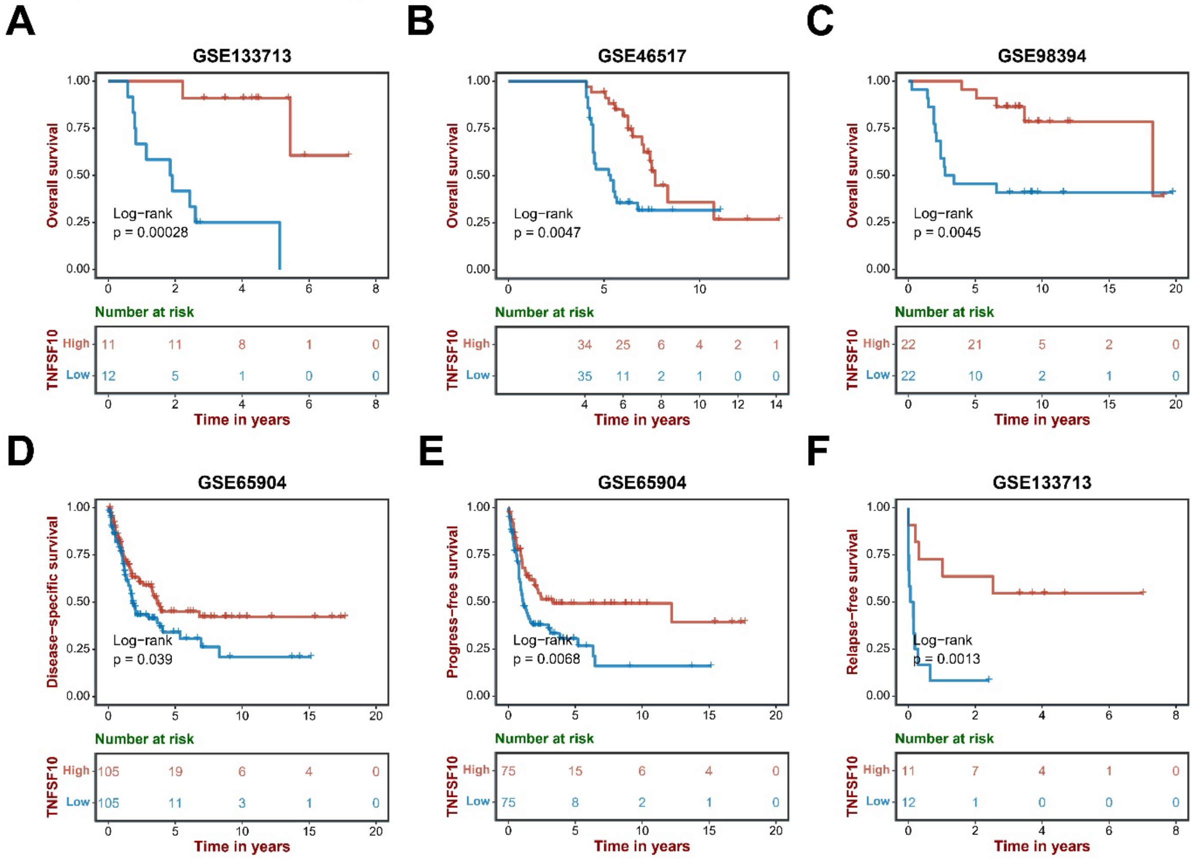

Supplementary Figure 2. Verification the value of TNFSF10 as a prognostic marker in SKCM. (A, B, C) The relationship between TNFSF10 and OS rate based on GSE133713, GSE46517, GSE98394. (D) The relationship between TNFSF10 and DSS rate based on GSE65904. (E) The relationship between TNFSF10 and progress-free survival based on GSE65904. (F) The relationship between TNFSF10 and relapse-free survival based on GSE133713.

Supplementary Figure 3. The expression of TNFSF10 in SKCM patients with different clinical features. (A) The expression of TNFSF10 in SKCM patients with different age levels. (B & C) The expression of TNFSF10 in SKCM patients with different pathological stages. (D) TNFSF10 expression in different cell types of GSE72056. (E) The expression of TNFSF10 in various cell types of SKCM patients of different genders.

Supplementary Figure 4

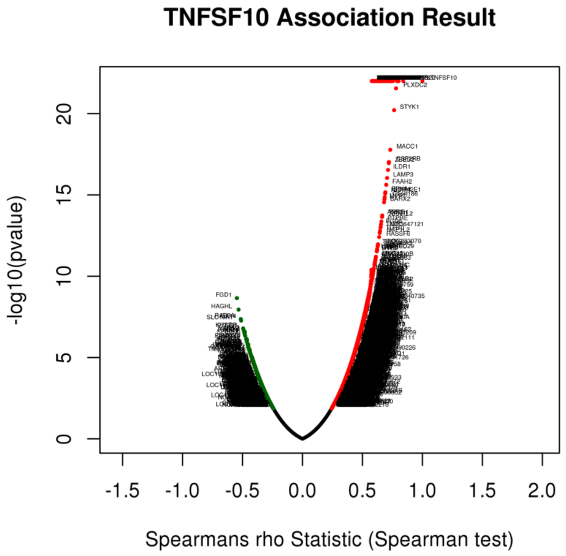

Supplementary Figure 4. Volcano plot showing genes related to TNFSF10.

Supplementary Figure 5

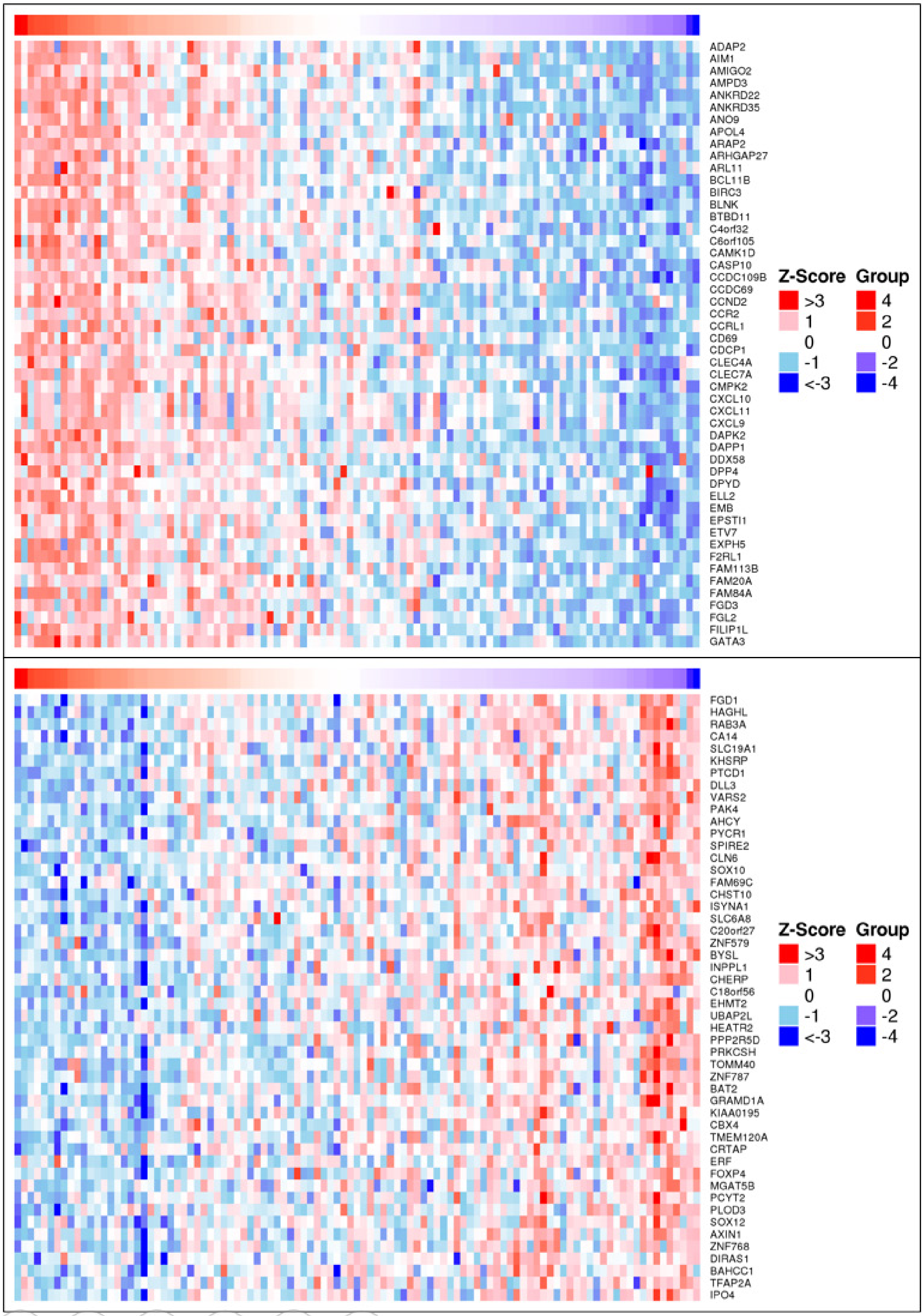

Supplementary Figure 5. Heatmaps showing the top fifty genes which are most positively/negatively related to TNFSF10.

Supplementary Figure 6

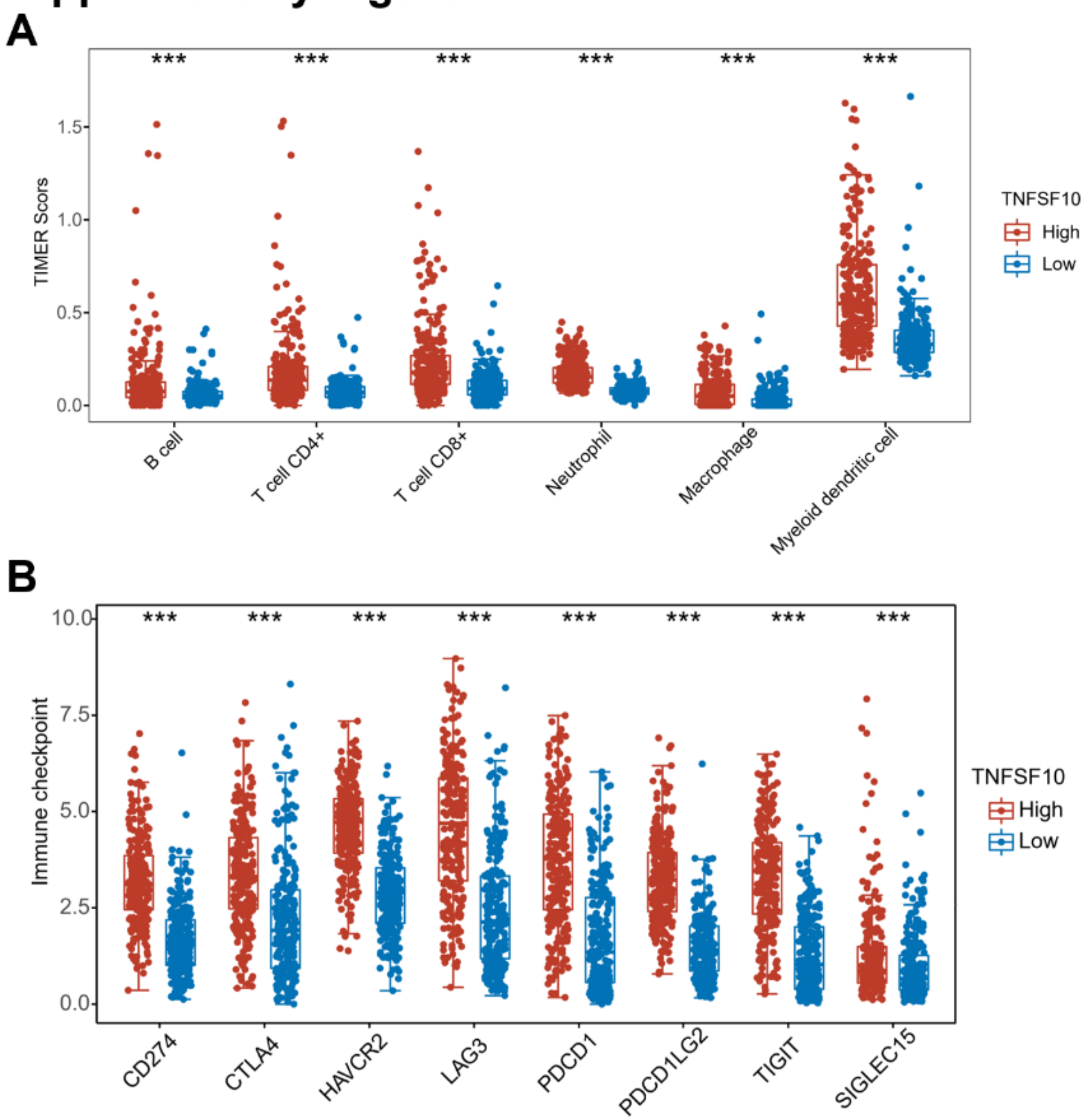

Supplementary Figure 6. Differences in immune cell scores and immune checkpoint genes of the high / low- expression groups of TNFSF10 in SKCM. (A) Differences in immune cell scores of the high /low- expression groups of TNFSF10 in SKCM. (B) Differences in immune checkpoint genes of the high / low- expression groups of TNFSF10 in SKCM.
